# Haptic Aesthetics and Bodily Properties of Ori Gersht’s Digital Art: A Behavioral and Eye-Tracking Study

**DOI:** 10.3389/fpsyg.2019.02520

**Published:** 2019-11-07

**Authors:** Marta Calbi, Hava Aldouby, Ori Gersht, Nunzio Langiulli, Vittorio Gallese, Maria Alessandra Umiltà

**Affiliations:** ^1^Unit of Neuroscience, Department of Medicine and Surgery, University of Parma, Parma, Italy; ^2^Department of Literature, Language, and the Arts, The Open University of Israel, Ra’anana, Israel; ^3^Department of Photography, University for the Creative Arts, Farnham, United Kingdom; ^4^Department of Art History Columbia University, Italian Academy for Advanced Studies, Columbia University, New York, NY, United States; ^5^Department of Food and Drug, University of Parma, Parma, Italy

**Keywords:** aesthetics, digital art, sensorimotor, behavioral, eye-tracking

## Abstract

Experimental aesthetics has shed light on the involvement of pre-motor areas in the perception of abstract art. However, the contribution of texture perception to aesthetic experience is still understudied. We hypothesized that digital screen-based art, despite its immateriality, might suggest potential sensorimotor stimulation. Original born-digital works of art were selected and manipulated by the artist himself. Five behavioral parameters: Beauty, Liking, Touch, Proximity, and Movement, were investigated under four experimental conditions: Resolution (high/low), and Magnitude (Entire image/detail). These were expected to modulate the quantity of material and textural information afforded by the image. While the Detail condition afforded less content-related information, our results show that it augmented the image’s haptic appeal. High Resolution improved the haptic and aesthetic properties of the images. Furthermore, aesthetic ratings positively correlated with sensorimotor ratings. Our results demonstrate a strict relation between the aesthetic and sensorimotor/haptic qualities of the images, empirically establishing a relationship between beholders’ bodily involvement and their aesthetic judgment of visual works of art. In addition, we found that beholders’ oculomotor behavior is selectively modulated by the perceptual manipulations being performed. The eye-tracking results indicate that the observation of the Entire, original images is the only condition in which the latency of the first fixation is shorter when participants gaze to the left side of the images. These results thus demonstrate the existence of a left-side bias during the observation of digital works of art, in particular, while participants are observing their original version.

## Introduction

The concept of haptic aesthetics has its foundations in the phenomenological insight that engaging with works of art involves more than vision alone (Marks, 2002; Sobchack, 2004; Paterson, 2012; Bruno, 2014; Gallese, 2018). The haptic, as an aesthetic term, emerged in late 19th and early 20th century German art-history, evolved in Walter Benjamin’s writings during the 1930s, and received a post-modernist twist in Deleuze and Guattari (1987). In particular, haptic (i.e., tactile and motor) aspects of engagement with moving-image art have been addressed by Marks (2002), Sobchack (2004), Barker (2009), and more recently Gallese and Guerra (2015, 2019). Marks describes haptic visuality as a ‘kind of seeing that uses the eye like an organ of touch,’ and Sobchack regards the film spectator as a ‘cinesthetic [*sic*] subject…able to commute seeing to touching and back again without a thought’ (Sobchack, 2004). More recently, Bruno focused on the ‘expanded spectatorial relations’ that arise in new-media art as a result of screen-related surface tensions (Bruno, 2014).

The abovementioned theoretical propositions have been corroborated by neurocognitive studies. As reviewed by Di Dio and Gallese (2009; see also Gallese and Di Dio, 2012), neuroaesthetics has demonstrated that experiencing a work of visual art involves sensorimotor and embodied affective processes. Evidence pertaining to the multimodal quality of perception highlighted a tight relationship between vision and touch (Keysers et al., 2004; Ebisch et al., 2008; Ebisch et al., 2014). Furthermore, experimental aesthetics is gradually shedding light on the involvement of pre-motor areas in the perception of abstract art (Freedberg and Gallese, 2007; Umiltà et al., 2012; Sbriscia-Fioretti et al., 2013). However, further investigation is still required with regard to the contribution of texture perception, and anticipation of touch, to aesthetic experience. Most studies in experimental aesthetics have focused on motor simulation, or motor empathy, driven by the functional mechanism of embodied simulation (Gallese, 2005, 2018). In this study we shift the view on aesthetic experience, considering it in terms of haptic engagement- where meaning and pleasure emerge through as-if (Damasio, 2010) tactile probing of the (virtual) surface. We approach aesthetic experience on the basis of its etymological derivation, from aisthêsis, generally understood as referring to sense perception (Paterson, 2012), or ‘sensuous perception’ (Brudzińska, 2010). As discussed by Brudzińska, and most pertinent to art-related experience, phenomenological theory understands aisthêsis as encompassing both immediate- or actual- perception, and ‘the experience of the possible,’ namely ‘a consciousness in the mode of the as if’ (Ebisch et al., 2008).

In the present study, we hypothesized that digital screen-based art, although essentially immaterial, might still suggest potential tactile stimulation, whether active (touching or desiring to touch) or passive (suggesting a sense of cutaneous stimulation). Fulfilling these conditions, the work of art could be regarded as haptically effective. This assumption is supported by studies showing that anticipation of touch is manifested in cortical networks that are primarily involved in either active or passive touch (Carlsson et al., 2000; Ebisch et al., 2008, 2014). To proceed in this vein, interrogation of art-related experience must pay particular attention to the role of such anticipations, i.e., either expecting to actively touch, or anticipating stimulation on the surface of the skin. This ‘haptic’ perspective may add depth to the already documented role of motor intentions in aesthetic experience.

Proximity, or as-if suggestion of proximity, is a pivotal trait of haptic vision and aesthetics. Paterson concludes that ‘from Riegl’s art history through Deleuze and Guattari, the haptic is consistently formulated in terms of closeness, of proximity’ (Paterson, 2012). In this experiment we focused on (1) the sense of proximity between the artwork and the participant’s body, and (2) the desire to touch the artwork, as behavioral indicators of the haptic effect. The experiment thus sought to assess haptic effectiveness, i.e., the capacity of particular aesthetic traits of the artworks to suggest potential tactile stimulation and induce a sense of proximity. In line with Bruno’s argument that, in the digital age, ‘materiality is not a question of materials but … of activating material relations’ (Bruno, 2014), we were seeking a particular view on how digital photography and video art might be re-inventing materiality today. The present experiment pursued indications for the ability of contemporary born-digital visual art to offer a range of tactile affordances. It also aimed to identify formal parameters that modulate these affordances and thereby affect viewers’ aesthetic pleasure. Inside this framework, eye movements were recorded while participants were contemplating the aesthetic and haptic properties embedded in digital works of art. Regarding the potential role of eye movements as a measure of aesthetic experience (Locher et al., 2007), an interesting and yet unanswered question regards the visual exploration pattern of digital artworks. In particular, the tendency to direct the gaze to the left side of digital works of art has not been investigated yet (e.g., Butler et al., 2005). Recent studies demonstrated the presence of leftward bias during scenes and artworks visual exploration (Ossandon et al., 2014; Rodway et al., 2019). It has been suggested that the left-gaze bias is due to the dominance of the right hemisphere for both emotional and configural processing (Christman and Hackworth, 1993; Bourne, 2008; Calvo and Nummenmaa, 2008; Innes et al., 2015).

The stimuli were original works of art provided by the London-based artist Ori Gersht. The artist was involved in all stages of stimuli preparation, including selection and manipulation of the images according to the experimental requirements. To our knowledge, this is the first study in experimental aesthetics deploying original, born-digital works of art as stimuli, thereby increasing the ecological validity of the study. Gersht’s art was elected as a case study because it presents a high degree of haptic effectiveness. In his art he deploys various strategies in order to augment tactile suggestiveness (Aldouby, in press). In preliminary discussions for the present experiment, the artist proposed that ‘with digital images … it’s as though the work of art needs to be somehow fetishized as an object, in order for the viewer to get the full experience.’ Reflecting on the assumptions underlying this study, Gersht further remarked: ‘It interests me if you can create the same desire to touch … as in encountering a sculpture or a painting. To achieve this … I feel that there needs to be something to the surface, to the feel of it, that can only be experienced through the eye, but something in us really pushes to want to experience it in a much more sensual, physical way.’ Our results, discussed below, appear to confirm the implicit intentions underlying Gersht’s art.

The stimuli were selected from four photographic series: *Blow Up* (2007), *Cells* (2013), *Love Me Love Me Not* (henceforth LMLMN, 2013), and *On Reflection* (2014). Except for LMLMN, Gersht created these series of still photographs either in conjunction or as an aftermath of video works (*Big Bang*, 2006; *Offering*, 2011; *On Reflection*, 2014). The stimuli were thus selected as accurate manifestations of the artist’s haptic aesthetic, albeit not in motion but in still form. The selected stimuli display a range of textures, from the deeply scarred wood of *Cell* to the texture of blood emulsified in milk in LMLMN.

The experiment registered five behavioral parameters: (1) desire to touch the image; (2) sense of proximity with the image; (3) movement detected in the image; (4) pleasure (liking); and (5) assessment of beauty. Of these, ‘proximity’ and ‘desire to touch’ were the most strongly related to haptic experience. The aesthetic appraisal questions were subdivided into “liking” and “beauty” based on previous demonstration that these two evaluations elicit different scores (van Paasschen et al., 2015; Mastandrea and Umiltà, 2016). The behavioral parameters were investigated in four experimental conditions: high and low resolution, entire image, and image detail. Marks (2000) phenomenological film theory hypothesizes that films which offer “more visual texture than the eye can apprehend, have the effect of overwhelming vision and spilling into other sense perceptions” (175). In the present study we manipulated the resolution of the stimuli, aiming to corroborate the assumption that enlarging the extent of visual information would indeed induce other modes of sensory engagement, beyond vision (Somaini and Casetti, 2018). Eye movements were analyzed in relation to each of the behavioral parameters and experimental conditions listed above. In sum, our study was aimed to combine eye gaze and explicit behavior during sensorimotor and aesthetic evaluations performed in response to the observation of digital works of art.

## Materials and Methods

### Participants

Thirty volunteers took part in the behavioral and eye-tracking study: 14 males, 16 females, mean age −*M* 24.2 [standard deviation (*SD)* = 2.1]. Of these, 20 participants were right-handed, nine ambidextrous and one was left-handed, as ascertained by the Edinburgh Handedness Inventory Oldfield (1971). All participants had normal or corrected-to-normal visual acuity and no history of neurological or psychiatric impairments. 11 participants had a left and 19 participants a right ocular dominance. Since one participant was discarded from eye tracking data analysis because of technical problems during the recording, the final sample for the eye tracking analysis consisted of 29 participants. The study has been conducted according to the principles expressed in the Declaration of Helsinki 2013. All participants provided a written informed consent to participate in the study, which was approved by the local ethical committee: Comitato Etico dell’Area Vasta Emilia Nord.

### Stimuli and Procedure

#### Stimuli

The stimuli consisted in color digital works of art created by the contemporary artist Ori Gersht. The artist himself selected eight digital works of art, considered by him the best ones for the aim of the study. Hence, stimuli were not reproductions, but real digital artistic productions. Each digital image was modified by the artist in order to be shown in four different experimental conditions: (1) Entire image; (2) Detail; (3) High Resolution; (4) Low Resolution. In this way, we had 32 digital images in total, eight for each condition (see Table 1).

**TABLE 1:** List of digital artworks selected by Ori Gersht and used as experimental stimuli.

**Entire image**		**Detail**	
**High**	**Low**	**High**	**Low**
Blow Up 01	Blow Up 01	Blow Up 12	Blow Up 12
Blow Up 04	Blow Up 04	Blow Up 13	Blow Up 13
Cell03_New	Cell03_New	Cell03Detail_New	Cell03Detail_New
Fusion_J03	Fusion_J03	Fusion_B05	Fusion_B05
Love Me Love Me Not_06	Love Me Love Me Not_06	Love Me Love Me Not_06	Love Me Love Me Not_06
Love Me Love Me Not_08	Love Me Love Me Not_08	Love Me Love Me Not_08	Love Me Love Me Not_08
Love Me Love Me Not_12	Love Me Love Me Not_12	Love Me Love Me Not_12	Love Me Love Me Not_12
Love Me Love Me Not_14	Love Me Love Me Not_14	Love Me Love Me Not_14	Love Me Love Me Not_14

By means of a Repeated-Measures ANOVA with two within-group factors (Resolution: High, Low; Magnitude: Entire image, Detail), we analyzed the differences in images mean dimension across conditions and the ANOVA did not reveal any significant result.

#### Apparatus

Digital images were shown on a 4K Ultra HD screen (28′′; 39.3 cm × 65.7 cm) with a luminance of 30 lumen and a resolution of 3840 × 2160 pixel, at a distance of 60 cm from participants. Tobii Pro Eye-Tracker X3-120 was used to record data on eye-movements with a sample frequency of 120 Hz. We used a double monitor setup: the eye tracker was mounted on the above described HD screen and it was connected to a laptop running Tobii Studio software 3.4.7, used as a secondary screen by the experimenter to check participants’ eye movements in real time and to record their answers (see below). After the classification of raw data as fixations by means of the I-VT Filter implemented in Tobii Studio, we extracted duration and latency of participants’ first fixations by means of homemade R scripts (R Core Team, 2019).

#### Procedure

The experimental task was preceded by participants’ assessment session: one day before the experiment, participants were asked to fill in several questionnaires via Google Forms. Participants’ art experience was estimated by the Art Experience Questionnaire (Chatterjee et al., 2010). Empathic traits were assessed through Interpersonal Reactivity Index (IRI; Davis, 1980).

Upon arrival in the laboratory, participants signed the informed consent form and after the assessment of manual and ocular dominance, read the instructions. After the evaluation of the correct position of the participant in front of the Tobii by means of the “track status meter,” a calibration procedure required participants to follow (without moving their head) a red bouncing ball, which paused at nine unpredictable positions on the screen, in a 3 × 3 configuration.

During the experimental task, participants were shown the 32 digital works of art (see above), each repeated six times, for a total of 192 trials. For each repetition, the image was associated with a different question: (1) “How much do you like it?” (2) “How beautiful is it?” (3) “How much do you want to touch it?” (4) “How close to you is the image?” (5) “How much movement do you perceive in the image?” (6) “How bright is the image?” (control condition) (All questions are translated from Italian). The six questions (experimental conditions) were presented in fully randomized order. Hence, experimental questions pertained to both aesthetic (questions 1 and 2) and sensorimotor judgment (questions 3–5). The control condition was used for a normalization procedure, see below. Participants were asked to answer verbally on a five points Likert scale, from 1 (not at all) to 5 (a lot) and, in order to avoid gaze shifts, the rating was recorded by the experimenter (e.g., Laukka et al., 2013; Savazzi et al., 2014). Each question was preceded by an instruction focusing participants’ attention on aspects of their aesthetic experience that would be the object of the subsequent experimental question. The six instructions were: (1) “Think about how much you like the next image”; (2) “Think about how beautiful is the next image”; (3) “Think about how much you want to touch the next image”; (4) “Think about how close to you is the next image”; (5) “Think about how much movement you perceive in the next image”; (6) “Think whatever you want about the next image” (control condition). There were two reasons for which we employed a condition in which there was no specific instruction given and asked participants a question related to brightness.

Firstly, we wanted to avoid any specific cognitive load during stimulus visual exploration in this condition and, secondly, we wanted to keep the structure of the trial identical to the experimental conditions. The question with regards to the brightness was a control condition and the related scores were used only for a normalization procedure. Our aim was to have a control condition as “neutral” as possible to be subtracted from the other experimental conditions.

**FIGURE 1:**
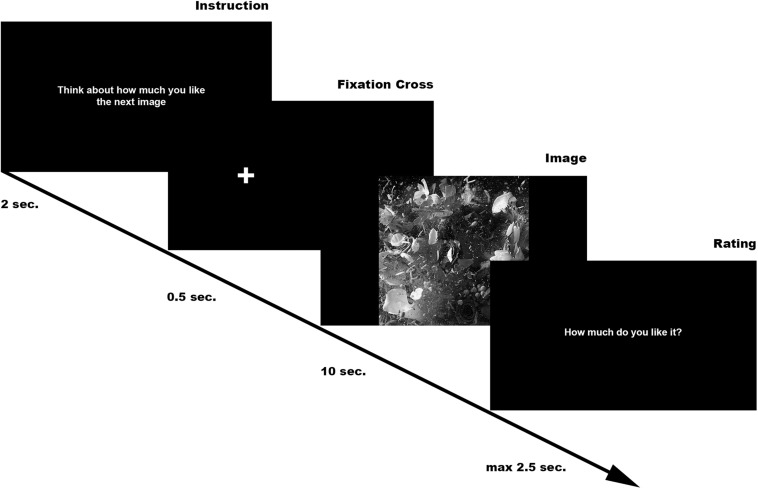
Experimental paradigm.

Specifically, each trial consisted of an Instruction, presented for 2 s, informing participants of the question they would have answered to at the end of the trial, followed by a white fixation cross on a black background of 0.5 s length (presented in balanced order on the left or on the right of the screen center to minimize central fixation bias (see Tatler, 2007; Guo and Shaw, 2015). The fixation cross was followed by the digital artwork presented for 10 s (for more details about presentation time see Brieber et al., 2014). At the end, participants were asked to rate the image: the question was presented for a maximum time of 2.5 s or until participant’s answer (Figure 1).

We built five different experimental sequences, each consisting of 192 differently randomized trials and of two breaks. The order of presentation of the sequences was counterbalanced across participants, each of them performing one out of the five sequences. Each trial had a length of 15 s and each sequence lasted about 48 min.

The experimental task was preceded by a training session that included two trials comprehending two images not pertaining to the experiment.

In order to maintain light-controlled conditions, the experiment was conducted in a dark room.

At the end of the experimental task, participants were asked to perform a familiarity test to ensure that they had never seen, prior to the experiment, the digital artworks used as stimuli.

#### Behavioral Analysis

To investigate whether digital works of art were capable of modulating aesthetic appreciation and sensorimotor evaluation of naïve beholders, behavioral data were analyzed by means of a cumulative link model for ordinal regression using R’s clm() function (see Christensen, 2019).

Ordinal regression is a maximum likelihood estimation within the logit model using model selected based on AIC. The convergence of model was assessed by inspecting the maximum absolute gradient of the log-likelihood function. The threshold was set to be equidistant from each adjacent value (see Christensen, 2019).

The model was obtained by means of hierarchical steps. To evaluate main effects in predicting outcome, the first step includes the predictors for measures of Question (five levels: Like, Beautiful, Touch, Proximity, Movement), Resolution (two levels: High, Low), and Magnitude (two levels: Entire, Detail). The second step includes interaction effects for ‘Question by Magnitude,’ ‘Question by Resolution,’ ‘Resolution by Magnitude.’ The third step includes the three-way interaction within predictors (see also Supplementary Table 1). Tukey’s test was used for *post hoc* comparisons among means.

The control condition was used for a normalization procedure: the average of the answer’s score given by each participant in this condition was subtracted from all the answers in the other conditions of the same participant.

In order to investigate the existence of a relation between participant’s immersive tendencies and their scores with the Likert scales, Spearman’s correlations were performed between behavioral scores for each of the five Questions, averaged across Resolution and Magnitude, and participants’ Fantasy-IRI scale converted in z-scores. In addition, the same averaged scores, obtained for each question, were correlated with those of each of all the other questions. Bonferroni correction for multiple comparisons was adopted.

#### Eye-Tracking Analysis

In order to investigate whether visual exploration patterns were modulated by the different experimental conditions and whether there was a lateralization bias, each image was divided into two identical and symmetrical Areas of Interests (AOIs), covering the whole image: Left and Right AOI. The latency of the first fixation, as well as the total number and duration of all fixations directed at each AOI, were analyzed by means of a linear mixed effects analysis, respectively. We started with a simple model and added parameters if their inclusion improved model fit. Likelihood ratio tests were used to establish whether the inclusion of random effects and interaction effects would significantly improve model fit. We entered each visual parameter as a dependent variable, and Question (five levels: Like, Beautiful, Touch, Proximity, Movement), Resolution (two levels: High, Low), Magnitude (two levels: Entire, Detail), and AOI (two levels: Left, Right) as independent fixed variables. We entered by participants intercept for the effect of AOI as random effects. For each parameter, the model was obtained by means of a hierarchical approach. For the three models, parameters were estimated using the full maximum likelihood (ML) procedure. Assumptions of normality and homoscedasticity were checked by inspecting the residuals of the models. Because of some deviations from normality, we additionally performed a bootstrapped estimate of fixed effects to further establish the significance of the predictors. A clustered bootstrap procedure with 10.000 bootstrap samples was employed and parametric 95% confidence intervals (CIs) were calculated for the parameter estimates (regarding fixed effects). We also tested the final three models using a more robust multilevel method that produced the same significant effects (for a similar analytical approach see Spruit et al., 2018) (see Supplementary Tables 2–7).

Tukey’s test was used for *post hoc* comparisons among means. For each visual parameter, the control condition was used for a normalization procedure: the average of the fixations made by each participant in this condition was subtracted from all the other fixations in the other conditions of the same participant.

For all analyses, we used R (R Core Team, 2019), lme4 (Bates et al., 2015; Lawrence, 2016; Lenth, 2016), and robustlmm (Koller, 2016).

## Results

### Behavioral Results

The results of the Art Experience Questionnaire showed an averaged score of 10,23 (*SD* = ± 7.81). IRI subscales averaged scores were the following: *Fantasy* 24,8 (*SD* = ± 5.66)*; Perspective-Taking* 25,4 (*SD* = ± 4.33); *Empathic Concern* 26,36 (*SD* = ± 3.27); *Personal Distress* 19,36 (*SD* = ± 5.04).

**FIGURE 2:**
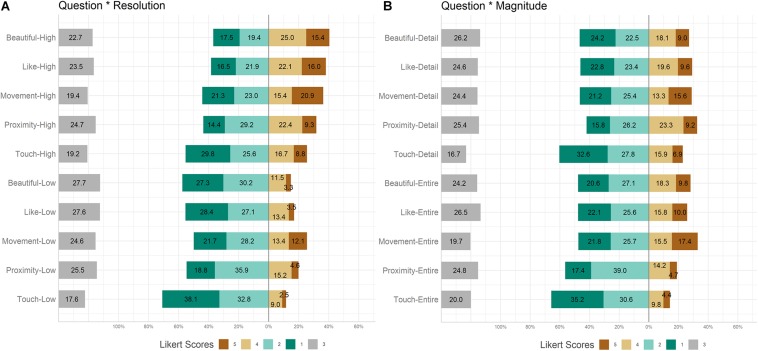
Behavioral results. **(A)** Question^∗^Resolution, stacked bar plot. **(B)** Question^∗^Magnitude, stacked bar plot.

The model revealed a main effect of Question [χ^2^(4) = 100.3, *p* < 0.001], with the score of the question related to the “desire to touch” significantly lower than that of all the other questions (all *ps* < 0.001). The model also revealed a main effect of Resolution [χ^2^(1) = 149.4, *p* < 0.001] with High Resolution images receiving higher ratings than Low Resolution images (*p* < 0.001). The model also revealed a main effect of Magnitude [χ^2^(1) = 6.3, *p* < 0.05] with Entire images receiving higher ratings than Detail images (*p* < 0.05). The model also revealed a main effect of ‘Question by Resolution’ [χ^2^(4) = 20.3, *p* < 0.001]: for all questions but Movement high resolution scores were significantly higher than low resolution (all *ps* < 0.01), showing that sensory motor effectiveness and aesthetic judgments were influenced by the higher extent of textural information available in the High Resolution stimuli. *Post hoc* tests showed that when stimuli were presented both in High and Low Resolution, Touch score was significantly lower than all the other scores (all *ps* < 0.01). When stimuli were presented in Low Resolution, Beautiful rating was lower than Movement rating (*p* < 0.05). Furthermore, the model showed a significant ‘Question by Magnitude’ interaction effect [χ^2^(4) = 14.6, *p* < 0.01]. Comparing the two Magnitudes, Detail images obtained higher scores for Proximity (*p* < 0.01), indicating that participants perceived the Detail images as closer than Entire images. Furthermore, considering both Entire and Detail images, Touch score was significantly the lowest among all the other questions (all *ps* < 0.01) (see Figure 2).

**FIGURE 3:**
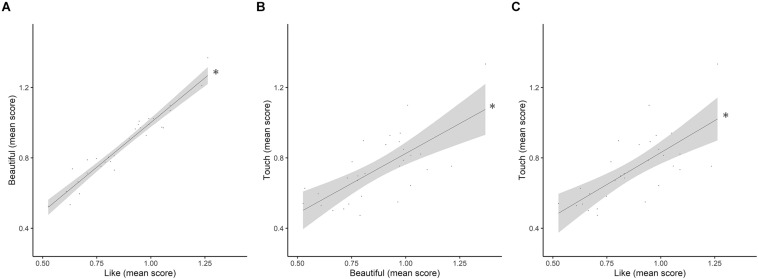
Correlations plots. **(A)** Correlation plot between Like and Beautiful mean normalized scores; **(B)** Correlation plot between Beautiful and Touch mean normalized scores; **(C)** Correlation plot between Like and Touch mean normalized scores. Gray area represents SE. ^∗^*p* < 0.05.

Spearman’s correlations were performed both between participants’ behavioral ratings to each Question and between behavioral ratings and Fantasy IRI scale. The results of the correlations performed with the data of the scores attributed to the five Questions correlated with each other, indicated that Like scores positively correlated with Beautiful (ρ = 0.94, *p* < 0.001), and with Touch scores (ρ = 0.71, *p* < 0.001) (see Figures 3A,C). The same analysis revealed that Beauty ratings positively correlated with Touch (ρ = 0.66, *p* < 0.001) (see Figure 3B).

The results of the correlations performed between behavioral scores to each Question and the IRI Fantasy subscale did not result significant.

### Eye-Tracking Results

#### Latency of First Fixations

**FIGURE 4:**
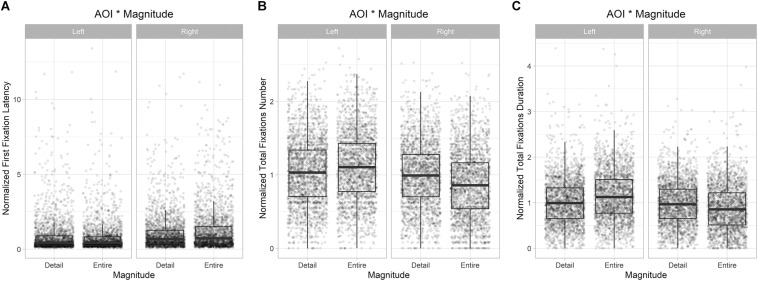
Eye-tracking results. **(A)** Normalized first fixations latency: AOI^∗^Magnitude significant interaction; **(B)** Normalized total fixations number: AOI^∗^Magnitude significant interaction. **(C)** Normalized total fixation duration: AOI^∗^Magnitude significant interaction.

The model explained 9.5% of the variance in latency taking into account the random effects ($R_{\text{m}}^{2}$ = 0.022; $R_{\text{c}}^{2}$ = 0.095). The model revealed a main effect of AOI (*F*_(__1_,_9114__)_ = 183, *p* < 0.001), with latency of first fixations directed at Left AOI on average being shorter than latency of first fixations directed at Right AOI. The model also revealed a significant ‘AOI by Magnitude’ interaction effect (*F*_(__1_,_9114__)_ = 24, *p* < 0.001). *Post hoc* tests showed that considering Entire images, latency of first fixations directed at Left AOI on average was shorter than latency of first fixations directed at Right AOI (*t*_(__64__)_ = −5, *p* < 0.001). The same difference was not significant when considering Detail images (*t*_(__64__)_ = −2.33, *p* > 0.05). By comparing the two magnitudes, *post hoc* tests showed that there was a significant difference in the Right AOI only: latency was shorter to Detail than to Entire images (Right: *t*_(__9143__)_ = −4.7, *p* < 0.001; Left: *t*_(__9143__)_ = 2.5, *p* = 0.06) (see Figure 4A).

#### Total Number of Fixations

The model explained 27% of the variance in total number of fixations taking into account the random effects ($R_{\text{m}}^{2}$ = 0.04; *$R_{\text{c}}^{2}$* = 0.27). The model revealed a main effect of AOI (*F*_(__1_,_9189__)_ = 228.2, *p* < 0.001), with total number of fixations directed at Left AOI on average being higher than fixations directed at Right AOI. The model also revealed a main effect of Resolution (*F*_(__1_,_27__)_ = 15.8, *p* < 0.001) with total number of fixations directed at High resolution images on average being higher than fixations directed at Low resolution images. The model also revealed a main effect of Magnitude (*F*_(__1_,_28__)_ = 12.8, *p* < 0.05): total number of fixations directed at Entire images on average was higher than fixations directed at Detail images. Furthermore, the model showed a significant ‘AOI by Magnitude’ interaction effect (*F*_(__1_,_9189__)_ = 121.5, *p* < 0.001). *Post hoc* tests showed that considering Entire images, total number of fixations directed at Left AOI on average was higher than total number of fixations directed at Right AOI (Entire: *t* = 4.05, *p* < 0.001). The same difference was not significant when considering Detail images (*t*_(__58__)_ = 0.6, *p* > 0.05). By comparing the two magnitudes, *post hoc* tests showed that, considering the Left AOI, participants made more fixations to Entire than to Detail images (*t*_(__9217__)_ = −6.8, *p* < 0.001), but when considering the Right AOI they made more fixations to Detail than to Entire images (*t*_(__9217__)_ = 11.1, *p* < 0.001) (see Figure 4B).

#### Total Duration of Fixations

The model explained 26% of the variance in total duration of fixations taking into account the random effects ($R_{\text{m}}^{2}$ = 0.04; $R_{\text{c}}^{2}$ = 0.26). The model revealed a main effect of AOI (*F*_(__1_,_9189__)_ = 204, *p* < 0.001), with total duration of fixations directed at Left AOI on average being longer than total duration of fixations directed at Right AOI. The model also revealed a main effect of Resolution (*F*_(__1_,_27__)_ = 27.16, *p* < 0.001) with total duration of fixations directed at High resolution images on average being longer than total duration of fixations directed at Low resolution images. The model also revealed a main effect of Magnitude (*F*_(__1_,_28__)_ = 9.05, *p* < 0.01): total duration of fixations directed at Entire images on average was longer than total duration of fixations directed at Detail images. Furthermore, the model showed a significant ‘AOI by Magnitude’ interaction effect (*F*_(__1_,_9189__)_ = 131.2, *p* < 0.001). *Post hoc* tests showed that considering Entire images, total duration of fixations directed at Left AOI on average was longer than total duration of fixations directed at Right AOI (*t*_(__58__)_ = 4.1, *p* < 0.001). This difference was not significant considering Detail images (*t*_(__58__)_ = 0.45, *p* > 0.05). By comparing the two magnitudes, *post hoc* tests showed that, considering the Left AOI, participants made longer fixations to Entire than to Detail images (*t*_(__9217__)_ = −11, *p* < 0.001), but when considering the Right AOI they made longer fixations to Detail than to Entire images (*t*_(__9217__)_ = 7.4, *p* < 0.001) (see Figure 4C).

## Discussion

In this study, we empirically investigated aesthetic experience by privileging the sensorimotor/haptic features of the experienced digital artworks. Of course, the here studied components of aesthetic experience are just one instantiation of the many levels at which images and artworks can be perceived and understood.

The first result that emerges from the analysis of behavioral data is that the two types of manipulation of the original images, Resolution and Magnitude respectively, had different effects on how participants rated stimuli in the different experimental conditions. Since these results are not just the unspecific outcome of generic changes to the stimuli, we must consider separately the effects of the two modifications made to the images.

An interesting finding about the ratings of digital works of art is that High-Resolution images received higher scores than the Low Resolution ones for all the questions, but the one related to movement. High-Resolution stimuli are identical to the original works of art made by the artist Ori Gersht, while Low Resolution stimuli have been adapted for the purpose of this experiment. As stated by Somaini and Casetti (2018), since resolution is an indicator of the quantity of detail contained in an image, controlling images’ resolution is a way of controlling their visibility. Lowering the resolution of an image is a way of reducing the access to its content, while presenting high resolution images increases values such as mimetic precision, sensory enhancement, immersive participation and artistic salience (Somaini and Casetti, 2018). According to this perspective, our results show that the observation of the original works of art by Ori Gersht establishes a bodily relationship between beholders and the aesthetic and haptic properties of the images.

Previous studies have shown the effect of manipulating works of art, both abstract and representational, but differently from our study, these previous studies were performed with the aim of investigating the ability to discriminate between the real works of art and their manipulated version (Gordon and Gardner, 1974; McManus et al., 1993; Locher et al., 1999). In our experiment, the highest rating values given to original works of art, that is, to the high resolution images, are in any case in agreement with all previous studies in which participants were asked whether they preferred original images of works of art or their modified versions (Locher et al., 2001; Leder et al., 2004; Di Dio et al., 2007).

Comparing the two Resolutions, movement was the only question that did not receive significantly different scores: participants did not perceive the images presented in different Resolutions as dissimilar in terms of their implied motion. This shows that the sense of implied movement is not affected by the experimental manipulation of image resolution. However, for the other four questions, Touch, Proximity, Liking, and Beautiful, the significant difference between the scores for high vs. low resolution supports our hypothesis that more visual information, pertaining to texture, color, and materiality in general, enhances participants’ sensory engagement and thereby the sense of presence.

Interestingly, comparing the two Magnitudes, Detail images obtained higher scores for the Proximity question, indicating that participants perceived the Detail images as closer to their body than Entire images. This result could be interpreted as a “zoom effect,” which is coherent with one of the purposes of the experiment, that is, to assess whether the additional information afforded by enlarged details of works of art amplifies the sense of closeness between the images and the participants’ bodies.

Within the framework of film theory, Gallese and Guerra (2015, 2019) suggested that close-ups play the crucial role of reinforcing the spectator’s haptic and tactile resonance with the image on the screen. Because enlargement of the image enhances its material and textural qualities, close-ups drive beholders’ attention on the material aspects of the object being filmed or photographed, thus evoking a greater experience of presence of the same object. According to this hypothesis, the magnification of the image would induce mechanisms of tactile engagement, enhancing the qualities of haptic detail, texture and the material consistency of the image. By means of enlargement, the bodily experience of digital works of art appears to be enhanced. Indeed, our results clearly demonstrate that participants perceived the details of Ori Gersht’s artworks as closer to their bodies when compared with whole images.

The results of a recent study of Heimann et al. (2014, 2019), aimed to investigate whether the brain of spectators responded differently to the observation of scenes filmed by different camera movements approaching the scene, are in full agreement with the present results and their interpretation. Heimann and colleagues demonstrated that dynamically reducing the distance from the observed scene enhances the activation of beholders’ sensorimotor cortex. Moreover, the same study showed that participants rated the movie clips in which the camera approached the scene as more bodily-involving than those filmed by a still camera.

The conditions of Resolution (high/low), and Magnitude (Entire image/detail) were expected to modulate the quantity of material and textural information afforded to viewers. Manipulating these conditions could thus be a useful strategy to investigate the hypothesis that haptic images compel viewers to process surplus information through other sensory faculties beside vision. The sensory overflow described by theoreticians of haptic aesthetics like Marks (2000, 2002) and Barker (2009), could result from excess of visual information calling forth other sense modalities beyond vision. In the present experiment, the conditions of Detail and High Resolution provided this surfeit of textural and material information. The results demonstrated that Detail images received the highest scores for Proximity, and that the desire to touch increased with the increase of resolution: although Touch scored the lowest among the behavioral questions (perhaps owing to a culturally ingrained inhibition about touching works of art), it is notable that the score was significantly higher in the condition of High Resolution, compared to the obverse condition of Low Resolution. In addition to the impact of Resolution on the desire to touch, it is notable that while the Detail condition afforded less content-related information, it appears to have augmented the observers’ feeling of proximity to the beheld images, recalling Mark Hansen’s notion of “the dynamic coupling of body and image” (Hansen, 2006). Our results seem to imply that the feeling of proximity to the beheld images might be correlated with aesthetic presence effect (Gumbrecht, 2004).

Furthermore, since Like and Beauty ratings positively correlated with Touch, this could indicate an explicit relationship between beholders’ body involvement (i.e., their sense of bodily presence), and the aesthetic judgment about the content of visual works of art. The present results support the role of embodied simulation in the observation of visual art, and indeed also highlight its role during aesthetic experience (Freedberg and Gallese, 2007; Di Dio and Gallese, 2009; Gallese and Di Dio, 2012; Umiltà et al., 2012; Sbriscia-Fioretti et al., 2013).

Our results which emerged from correlation analyses demonstrated that the more the participants gave high scores to the two aesthetic judgments, the more they wanted to touch the presented works of art, or vice-versa: the sense of tactile arousal might have augmented participants’ aesthetic pleasure. Noticeably, similar results were recently obtained by Siri et al. (2018). These authors demonstrated a significant correlation between participant’s tendency to identify with the emotional states of fictional characters and the rating scores of explicit aesthetic judgment and desire to touch, both attributed to abstract works of art. Although we did not find any significant correlations with the IRI Fantasy subscale, the results of both studies indicate a relation between participants’ explicit aesthetic evaluation and their will to actively interact with the observed works of art. Moreover, our results seem to imply that the feeling of proximity to the beheld images might be correlated with aesthetic presence effect (Gumbrecht, 2004). To draw on W. J. T. Mitchell’s seminal question: *What Do Pictures Want?* (Mitchell, 2005), which implies both lack and desire, our results indicate the crucial role of texture and material information in restoring to digital pictures the body that they “want.”

Our experimental paradigm gave us the opportunity to study conditions in which the same visual stimuli were freely explored while participants were involved in different evaluative aesthetic and sensorimotor experiences. We found that beholders’ oculomotor behavior, as revealed by eye-tracking, selectively changed in relation to the different manipulations of the works of art, although it did not change in relation to the different questions. The crucial role of Magnitude in the modulation of gaze latency is demonstrated by the fact that the first fixation was faster only when it was performed on the left side of Entire images. Most models of overt visual search involving multi featured stimuli, suggest two phases (Holmes and Zanker, 2012): “The first being associated with scanning the visual field for relevant locations, and the second being associated with the more detailed identification, comparison, and evaluation of the different regions of the image. The first phase has been shown to be highly susceptible to the distribution of low-level image features over the visual field, and is characterized by frequent, short fixations. The second phase introduces top–down effects, for example, driven by the task being performed, the emotional content of the image, or personal preferences and it results in fewer but longer fixations” (Holmes and Zanker, 2012).

Our results showed that the latency of the first fixation was not modulated by the participants’ task. Thus, our eye-tracking data do not seem to be in accord with the above mentioned dichotomous view between a first bottom-up phase of visual exploration, guided by a purely perceptual mechanism, and a second phase controlled by top–down mechanisms. In contrast, the occurrence of a left-side bias for the first fixation in terms of latency, suggests the coexistence of perceptual, sensorimotor and aesthetic experiences, since in our free-viewing paradigm, participants were instructed in advance about which of the behavioral tasks to be engaged with during the subsequent image presentation, explicitly asking them to actively focus their attention on the aesthetic, haptic or sensorimotor experiences they were having while observing the works of art, respectively.

Our study for the first time demonstrates the existence of a left-side bias during the observation of digital works of art. In particular, the left-side bias occurred in terms of total fixations number and duration and first fixations latency during the observation of Entire works of art. It is possible to speculate that the feeling of presence induced by participants’ focusing on the complexity and the completeness of the beheld original images, might have been accompanied by a sort of emotional arousal, thus leading to a predominant activation of the right hemisphere, leading in turn to the observed left-side bias in their oculomotor exploration of those same images. Many studies demonstrated the presence of a left-side bias when processing the facial expression of emotions, interpreting these results as due to the specialization of the right hemisphere in emotion processing (Christman and Hackworth, 1993; Bourne, 2008; Calvo and Nummenmaa, 2008; Innes et al., 2015). Recently, in accord with our results, Rodway et al. (2019) demonstrated the occurrence of a leftward bias for the judgment of the attractiveness of abstract works of art.

It remains for future investigations to determine whether haptic effectiveness, and presence effect, might be graded in empirical parameters. Applying these insights to contemporary art may shed light on the growing quest for presence in an age of increasing uncertainty.

### Constraints on Generality (COG) and Limits

Indeed, our study employed digital works of art belonging to only one artist and thus our results need further studies in order to generalize them from digital artist to ‘digital art’ as such. We have a case-study that for the first time addresses from an empirical scientific perspective the engagement with specific contemporary real digital works of art, so that the artist is a co-author of the study. It’s a pioneering study that paves the road to future generalizations, through more empirical work. In accordance with the statement of constraints of generality (Simons et al., 2017, p. 1126), we hypothesize that future experiments employing digital artworks belonging to other artists, manipulated as we have in this study (Magnitude and Resolution), will give similar results. We have no reason to believe that the results depend on other characteristics of the participants or context. A limit of the study is the lack of comparison between artistic and non-artistic digital objects. However, constructing control stimuli comparable with the artistic ones in terms of perceptual features is often challenging and not always possible. Nonetheless, manipulation of the original artworks in terms of Resolution and Magnitude may “transform” them into non-artistic objects.

## Data Availability Statement

The raw data supporting the conclusions of this manuscript are available on Zenodo repository (https://doi.org/10.5281/zenodo.3524128).

## Ethics Statement

The studies involving human participants were reviewed and approved by Comitato Etico dell’Area Vasta Emilia Nord. Participants provided their written informed consent to participate in this study.

## Author Contributions

MC, HA, OG, VG, and MU designed the experiment. MC and NL performed data acquisition and analyses. MC, HA, NL, VG, and MU interpreted the results. MU, HA, and MC wrote the manuscript. All authors have contributed to, seen and approved the manuscript.

## Conflict of Interest

The authors declare that the research was conducted in the absence of any commercial or financial relationships that could be construed as a potential conflict of interest.
