# Supplementary material for: Haptic Aesthetics and Bodily Properties of Ori Gersht’s Digital Art: A Behavioral and Eye-Tracking Study
Source: Front Psychol. 2019 Nov 7;10:2520. doi: 10.3389/fpsyg.2019.02520 (PMC6853892; doi:10.3389/fpsyg.2019.02520)
Supplement: Supplementary file 1 [file Data_Sheet_1.PDF]

Supplementary Material

Haptic aesthetics and bodily properties of Ori Gersht's digital art: a behavioral and eye-tracking study.

Marta Calbi*, Hava Aldouby, Ori Gersht, Nunzio Langiulli, Vittorio Gallese, Maria Alessandra Umiltà*

* Correspondence:

Marta Calbi: calbimarta@gmail.com - marta.calbi@unipr.it

Maria Alessandra Umiltà: mariaalessandra.umilta@unipr.it

1 Supplementary Tables

Behavioral					
ordinal::clm(Scores ~ Question + Resolution + Magnitude + Question:Resolution + Question:Magnitude, data)					
	df	***AIC***	***Log-Lik***	χ^2	***p value***
**Reduced model (Null Model)**	2	14920.79	-7458.4	-	-
**Question**	6	14830.54	-7409.3	98.269	*p* <.001
**Resolution**	7	14681.33	-7333.7	151.215	*p* <.001
**Magnitude**	8	14676.55	-7330.3	6.783	*p* <.01
**Question:Resolution**	12	14664.54	-7320.2	20.046	*p* <.001
**Question:Magnitude**	16	14657.94	-7312.9	14.647	*p* <.001

Supplementary Table 1: Detailed description of nested models for behavioral scores. *Df* = degrees of freedom of the model, *AIC* = Akaike Information Criterion, R^2_m=marginal R^2, R^2_c=conditional R^2, *Log-Lik* = Log Likelihood ratio test, χ2 = Chi-squared.

Latency of first fixation								
lme4::lmer (Latency ~ AOI + AOI:Magnitude + (1|AOI:Participants), data)								
	df	*AIC*	*BIC*	R^2_m	R^2_c	*Log-Lik*	χ^2	*p value*
**Reduced model (Null Model)**	2	29542.21	29556.46	-	-	-14769	-	-
**AOI**	3	29358.97	29380.35	0.02	-	-14676	185.24	*p* <.001
**AOI:Magnitude**	5	29336.98	29372.61	0.022	-	-14664	26	*p* <.001
**Rand. Intercept Participants*AOI**	6	28819.82	28862.58	0.022	0.095	-14404	519.16	*p* <.001

Supplementary Table 2: Detailed description of nested models for latency of first fixation. *Df* = degrees of freedom of the model, *AIC* = Akaike Information Criterion, *BIC* = Bayeasian Information Criterion, R^2_m=marginal R^2, R^2_c=conditional R^2, *Log-Lik* = Log-Likelihood ratio test, χ^2 = Chi-squared.

Total number of fixations								
lme4::lmer(Number of Fix.~ AOI + Resolution + Magnitude + AOI:Magnitude + (1|AOI: Participants), data)								
	df	*AIC*	*BIC*	R^2_m	R^2_c	*Log-Lik*	χ^2	*p value*
**Reduced model (Null Model)**	2	11932.01	11946.28	-	-	-5964	-	-
**AOI**	3	11703.74	11725.14	0.024	-	-5848.9	230.27	*p* <.001
**Resolution**	4	11694.27	11722.81	0.025	-	-5843.1	11.47	*p* <.001
**Magnitude**	5	11689.35	11725.02	0.025	-	-5839.7	6.92	*p* <.01
**AOI:Magnitude**	6	11571.01	11613.81	0.038	-	-5779.5	120.34	*p* <.001
**Rand. Intercept Participants*AOI**	7	9274.26	9324.2	0.038	0.27	-4630.1	2298.7	*p* <.001

Supplementary Table 3: Detailed description of nested models for total number of fixations. df= degrees of freedom of the model, AIC=Akaike Information Criterion, BIC=Bayeasian Information Criterion, R^2_m=marginal R^2, R^2_c=conditional R^2, Log-Lik = Log-Likelihood ratio test, χ^2 = Chi-squared.

Total duration of fixations								
lme4::lmer(Durution of Fix. ~ AOI + Resolution + Magnitude + AOI:Magnitude + (1|AOI:Participants), data)								
	df	**AIC**	**BIC**	R^2_m	R^2_c	**Log-Lik**	χ^2	*p* **value**
**Reduced model (Null Model)**	2	14116.18	14130.45	-	-	-7056.1	-	-
**AOI**	3	13911.57	13932.98	0.021	-	-6952.8	206.6	*p* <.001
**Resolution**	4	13899.57	13928.1	0.023	-	-6945.8	14	*p* <.001
**Magnitude**	5	13896.77	13932.44	0.023	-	-6943.4	4.8	*p* <.05
**AOI:Magnitude**	6	13768.82	13811.62	0.037	-	-6878.4	129.9	*p* <.001
**Rand. Intercept Participants*AOI**	7	11634.46	11684.39	0.036	0.26	-5810.2	2136.37	*p* <.001

Supplementary Table 4: Detailed description of nested models for total duration of fixations. df= degrees of freedom of the model, AIC=Akaike Information Criterion, BIC=Bayeasian Information Criterion, R^2_m=marginal R^2, R^2_c=conditional R^2, Log-Lik = Log-Likelihood ratio test, χ^2 = Chi-squared.

Latency of first fixation							
	**LMER**				**RLMR**		
**Fixed Effects**	***β***	***SE***	***t***	***CI***	**β**	***SE***	***t***
Intercept	0.84	0.065	12.89	[0.71,0.97]	0.64	0.043	14.64
AOIright	0.21	0.092	2.33	[0.03,0.39]	0.21	0.061	3.4
AOILeft:MagnitudeFull	-0.08	0.034	-2.5	[-0.15,-0.02]	-0.03	0.02	-1.55
AOIRight:MagnitudeFull	0.16	0.034	4.74	[0.09, 0.23]	0.07	0.02	3.64

Supplementary Table 5: Fixed effects compared between robust estimation of Linear Mixed Model (RLMR) and the estimation method implemented in LME4. *β* = estimate, *SE* = standard error of estimate, *t* = t-value, *CI* = 95 % bootstrapped confidence interval (10.000 bootstrap samples was employed).

Total number of fixations							
	**LMER**				**RLMR**		
**Fixed Effects**	**β**	***SE***	***t***	***CI***	**β**	***SE***	***t***
Intercept	1.05	0.043	24.5	[0.96,1.13]	1.04	0.047	22.25
AOIright	-0.04	0.06	-0.63	[-0.16,0.08]	-0.034	0.066	-0.53
ResolutionLow	-0.03	0.008	-3.92	[-0.05,-0.02]	-0.03	0.008	-3.55
MagnitudeFull	0.08	0.011	6.78	[0.05,0.10]	0.08	0.012	7.05
AOIRight:MagnitudeFull	-0.21	0.016	-12.63	[-0.24,-0.17]	-0.22	0.016	-13.45

Supplementary Table 6: Fixed effects compared between robust estimation of Linear Mixed Model (RLMR) and the estimation method implemented in LME4. *β* = estimate, *SE* = standard error of estimate, *t* = t-value, *CI* = 95 % bootstrapped confidence interval (10.000 bootstrap samples was employed).

Total duration of fixations							
	**LMER**				**RLMR**		
**Fixed Effects**	**β**	***SE***	***t***	***CI***	**β**	***SE***	***t***
Intercept	1.03	0.047	22.05	[0.94,1.12]	1.012	0.05	20.43
AOIright	-0.03	0.067	-0.45	[-0.16,0.1]	-0.02	0.07	-0.3
ResolutionLow	-0.04	0.009	-4.29	[-0.06,-0.02]	-0.04	0.009	-4.65
MagnitudeFull	0.14	0.013	10.97	[0.12,0.17]	0.15	0.013	11.42
AOIRight:MagnitudeFull	-0.24	0.018	-13.01	[-0.28,-0.20]	-0.25	0.018	-14.04

Supplementary Table 7: Fixed effects compared between robust estimation of Linear Mixed Model (RLMR) and the estimation method implemented in LME4. *β* = estimate, *SE* = standard error of estimate, *t* = t-value, *CI* = 95 % bootstrapped confidence interval (10.000 bootstrap samples was employed).
